# Nonlocal correlations transmitted between quantum dots via short topological superconductor

**DOI:** 10.1038/s41598-024-64578-4

**Published:** 2024-06-15

**Authors:** G. Górski, K. P. Wójcik, J. Barański, I. Weymann, T. Domański

**Affiliations:** 1 Institute of Physics, College of Natural Sciences, University of Rzeszów, 35-310 Rzeszów, Poland; 2 Institute of Molecular Physics, Polish Academy of Sciences, 60-179 Poznań, Poland; 3 Polish Air Force University, ul. Dywizjonu 303 nr 35, 08-521 Dȩblin, Poland; 4 Institute of Spintronics and Quantum Information, Faculty of Physics, Adam Mickiewicz University, 61-614 Poznań, Poland; 5 Institute of Physics, Maria Curie-Skłodowska University, 20-031 Lublin, Poland

**Keywords:** Nanoscale devices, Superconducting devices, Condensed-matter physics, Topological matter

## Abstract

We study the quasiparticle states and nonlocal correlations of a hybrid structure, comprising two quantum dots interconnected through a short-length topological superconducting nanowire hosting overlaping Majorana modes. We show that the hybridization between different components of this setup gives rise to the emergence of molecular states, which are responsible for nonlocal correlations. We inspect the resulting energy structure, focusing on the inter-dependence between the quasiparticles of individual quantum dots. We predict the existence of nonlocal effects, which could be accessed and probed by crossed Andreev reflection spectroscopy. Our study would be relevant to a recent experimental realization of the minimal Kitaev model [T. Dvir *et al.*, Nature **614**, 445 (2023)], by considering its hybrid structure with side-attached quantum dots.

## Introduction

Majorana quasiparticles^1,2^, emerging at the boundaries of topological superconductors, are currently a topic of intensive studies, motivated by perspectives of using them as stable quantum bits (immune to decoherence due to topological protection) and in quantum computing (owing to their non-Abelian character)^3^. Experimental signatures of the zero-energy Majorana modes have been reported in numerous systems, ranging from (i) semiconducting nanowires and/or nanochains of magnetic atoms contacted with conventional superconductors, (ii) interfaces of the planar Josephson junctions or (iii) outer boundaries of magnetic islands deposited on superconducting surfaces [see Refs.^4–7^ for a comprehensive overview]. Another route for obtaining the Majorana modes is possible in vortices of triplet superconductors. Theoretical and experimental advancements in this direction include e.g. topological insulator/s-wave superconductor heterostructures^8,9^ and intrinsic superconducting topological insulators, such as FeTeSe^10–14^. Majorana modes have been also predicted to emerge in more complex magnetic textures proximitized to superconducting materials, such as those of the skyrmion geometry. In all of these platforms the Majorana quasiparticles do always appear in pairs. It has not been firmly established, however, whether they are mutually correlated over certain spatial or temporal scales. Some consequences caused by a hybridization of the Majorana modes confined in nearby vortices have been so far discussed in Refs^15–17^, inspecting extent of their coherence.

A convenient (although indirect) method for probing the interdependence of the Majorana boundary modes is to exploit hybrid nanostructures, such as those composed of quantum dots side-coupled to topological superconductors. Leakage of the Majorana modes into these objects has been predicted theoretically^18^ and confirmed experimentally^19^. Such approach has stimulated further experimental studies^20^, inspecting the nonlocality of emergent Majorana modes hybridized with quantum dot states. From the characteristic behavior of their energy spectra, associated with splittings and anticrossings, it is possible to extract the degree of nonlocality of Majorana quasiparticles quantified by the spin canting angle, as discussed in the next section. Such canting can be manifested either by a partially separated Andreev bound state or by a nonlocal state consistent with the well-developed Majorana mode.

**Figure 1:**
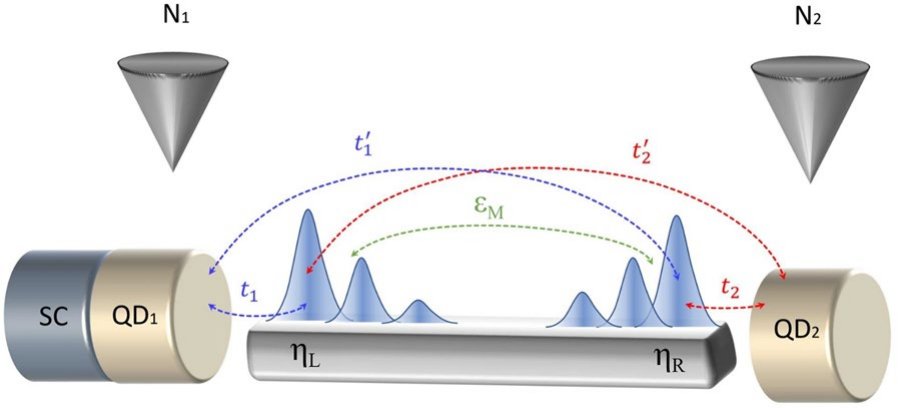
Schematic of the considered hybrid nanostructure, consisting of two quantum dots (QD_1,2_) attached to the opposite sides of the superconducting topological nanowire, hosting Majorana modes described by the operators $\eta _{\rm{L}}$ and $\eta _{\rm{R}}$. The electronic spectrum of QD_1_ can be probed by the charge transport measurements imposed by the bias voltage in the N_1_-QD_1_-SC part of this setup.

To address the problem of nonlocality, here we propose to consider a hybrid nanostructure consisting of two quantum dots interconnected via the topological superconductor. More specifically, we focus on the nonlocal correlations transmitted between these quantum dots solely through the short-length topological superconductor, allowing for finite overlap of the Majorana wave-functions (see Fig. 1). The considered setup would be particularly relevant for the minimal-length nanowires, such as realized recently Kitaev chain comprising only two or three sites^21,22^. In such short wires, the Majorana modes indeed do overlap with one another, so their leakage onto any side-attached quantum dot(s) would be a source of mutual cross-correlations^23^. Here we identify signatures of the inter-dot correlations suitable for experimental detection via the crossed Andreev spectroscopy.

Our study reveals that the leakage of Majorana modes into the quantum dots induces a nonlocal electron pairing, both in the triplet and singlet channels. Furthermore, the quasiparticle states of the system are showing up simultaneously in both quantum dots, although with different spectral weights, strongly dependent on the canting angle. Such nonlocal pairings can induce crossed Andreev reflections, empirically indicating distant cross-correlations. We determine transmittance of the local and nonlocal charge transport channels operating in the subgap region, which provide information about the conventional and topological quasiparticles of this hybrid nanostructure. We believe that our findings shall foster further endeavours in the *bottom-up* engineering of topological superconducting nanowires, such as these reported in Refs.^21,22^.

## Model

The considered hybrid structure, as displayed in Fig. 1, can be described by the following microscopic Hamiltonian

1 $$H = H_{\rm{MW}} + H_{\rm{L}} + H_{\rm{R}},$$

where $H_{\rm{MW}}$ models the Majorana wire (MW) attached to the left ($H_{\rm{L}}$) and right ($H_{\rm{R}}$) quantum dot junctions, respectively. We assume that the left part is formed by the first quantum dot (QD_1_), which is weakly coupled to a normal metallic lead (N_1_) and strongly hybridized with conventional (*s*-wave) superconductor (SC). Under such conditions, the superconducting proximity effect gives rise to the formation of trivial (finite-energy) bound states at QD_1_^24–27^. Furthermore, coupling QD_1_ to the topological superconductor allows the Majorana mode to leak into this quantum dot, and by interfering with the trivial bound states, such subsystem develops joint (‘molecular’) quasiparticle spectrum. We will examine in detail the corresponding spin-resolved energy structure of QD_1_ junction, which can be formally described by

2 $$\begin{aligned} H_{\rm{L}}{} & {} = \sum _{\sigma } \varepsilon _1 d_{1\sigma }^\dag d_{1\sigma } +\sum _{\textbf{k}\sigma }\varepsilon _{{N_1}{\textbf{k}}} c^\dag _{{N_1}{\textbf{k}}\sigma } c_{{N_1}{\textbf{k}}\sigma } +\sum _{{\textbf{k}}\sigma }\varepsilon _{{\rm{S}}{\textbf{k}}} c^\dag _{{\rm{S}}{\textbf{k}}\sigma } c_{{\rm{S}}{{\textbf{k}}}\sigma }- \Delta _{S}\sum _{{\textbf{k}}} ( c^{\dagger }_{{\rm{S}}{\textbf{k}}\uparrow }c^{\dagger }_{{\rm {S}}-{\textbf{k}} \downarrow }+\text {H.c.}) \nonumber \\{} & {} \quad +\sum _{{\textbf{k}}\sigma } \sum _{\beta ={{N_1,S}}} V_{\beta \sigma } \left( d^\dag _{1 \sigma } c_{\beta {\textbf{k}}\sigma } + c^\dag _{\beta {\textbf{k}}\sigma } d_{1 \sigma } \right) . \end{aligned}$$

here the operator $d_{1 \sigma }$ ($d^\dag _{1 \sigma }$) annihilates (creates) an electron with spin $\sigma$ and energy $\varepsilon _1$ at QD_1_, the operators $c_{\beta {\textbf{k}}\sigma }$ ($c^\dag _{\beta {\textbf{k}}\sigma }$) correspond to itinerant electrons with spin $\sigma$ and momentum $\textbf{k}$ in external electrodes $\beta =\{{\rm{N}}_1,{\rm{S}}\}$, while $\varepsilon _{{\beta }{\textbf{k}}}$ denotes the energy of respective electrons and $\Delta _{S}$ stands for the isotropic pairing gap. The last term in Eq. (2) describes the hybridization between the mobile electrons of the leads and QD_1_ with the corresponding tunneling matrix elements $V_{\beta \sigma }$.

On the other hand, the opposite-side quantum dot (QD_2_) is assumed to be weakly coupled to another normal lead (N_2_), and can be modelled by

3 $$\begin{aligned}{} & {} H_{\rm{R}} = \sum _{\sigma } \varepsilon _2 d_{2\sigma }^\dag d_{2\sigma } +\sum _{{\textbf{k}}\sigma }\varepsilon _{{N_2}{\textbf{k}}} c^\dag _{{N_2}{\textbf{k}}\sigma } c_{{N_2}{\textbf{k}}\sigma } +\sum _{{\textbf{k}}\sigma } V_{{N_2}\sigma } \left( d^\dag _{2 \sigma }c_{{N_2}{\textbf{k}}\sigma } + c_{{N_2}{\textbf{k}}\sigma }^\dag d_{2 \sigma } \right) , \end{aligned}$$

with standard notation for the electron operators. We assume that the distance between QD_1_ and QD_2_ is large enough for their direct coupling to be negligible. Thus, any cross-correlations induced between the quantum dots will be transmitted by the topological superconducting nanowire.

Finally, the low-energy Hamiltonian of the Majorana nanowire can be expressed as^28^

4 $$\begin{aligned} H_{\rm{MW}} = \sum _{r=1,2}\sum _{\alpha ={\rm{L,R}}} \sum _\sigma (\lambda _{r\sigma }^{\alpha }d^\dag _{r\sigma } \eta _\alpha + {\lambda _{r\sigma }^{\alpha *}} \eta _\alpha d_{r\sigma }) + i \varepsilon _M \eta _{\rm{L}} \eta_{\rm{R}}, \end{aligned}$$

where $\eta _\alpha =\eta _\alpha ^\dag$ are the self-conjugated Majorana operators, $\varepsilon _M$ stands for an overlap between the Majorana modes, while $\lambda _{r\sigma }^{\alpha }$ denotes the hybridization of $\alpha$-MBS (Majorana bound state) to QD_r_, as illustrated in Fig. 1.

It is worth to emphasize, that the Majorana modes hybridize with the side-attached quantum dots, depending on their spin^20,29–31^. This is practically caused by magnetic fields or magnetic textures, which are necessary to induce the inter-site triplet pairing in order to allow for topological transition. To capture such effect, we introduce the following spin-dependent couplings^29^

5 $$\begin{aligned} \begin{array}{ll} \lambda _{1\uparrow }^{L}=\sqrt{2}t_{1}\sin \frac{\theta }{2}, &{} \hspace{2cm} \lambda _{1\uparrow }^{R}=-i\sqrt{2}t'_{1} \sin \frac{\theta }{2}, \\ \lambda _{1\downarrow }^{L}=-\sqrt{2}t_{1} \cos \frac{\theta }{2}, &{}\hspace{2cm} \lambda _{1\downarrow }^{R}=-i\sqrt{2}t'_{1} \cos \frac{\theta }{2}, \end{array} \end{aligned}$$

where $t_r$ ($t_r'$) denotes the tunnel matrix elements between a given quantum dot and neighboring (distant) Majorana mode, while $\theta$ stands for the spin canting angle characterizing QD_1_-topological nanowire hybrid structure. Similarly for the other dot,

6 $$\begin{aligned} \begin{array}{ll} \lambda _{2\uparrow }^{L}=\sqrt{2}t'_{2}\sin \frac{\theta }{2}, &{} \hspace{2cm} \lambda _{2\uparrow }^{R}=-i\sqrt{2}t_{2} \sin \frac{\theta }{2}, \\ \lambda _{2\downarrow }^{L}=-\sqrt{2}t'_{2} \cos \frac{\theta }{2}, &{}\hspace{2cm} \lambda _{2\downarrow }^{R}=-i\sqrt{2}t_{2} \cos \frac{\theta }{2}, \end{array} \end{aligned}$$

as illustrated in Fig. 1.

Since each quantum dot is also coupled to continuum fermionic states of its own metallic electrode, the in-gap states acquire finite life-times (for details see Ref.^26^). In the wide bandwidth limit, the resulting broadening is given by the coupling strength $\Gamma _{{\rm{N}}_r\sigma } = \pi \rho _r |V_{{\rm{N}}_r\sigma }|^2$, where $\rho _r$ is the density of states of the given lead, which is assumed to be constant inside the pairing gap of superconductor. In addition, the first quantum dot is coupled to the superconducting electrode with coupling strength $\Gamma _S = \pi \rho _S |V_S|^2$, where $\rho _S$ is the density of states of superconductor in the normal state. For specific calculations, we also impose the spin-independent couplings, $\Gamma _{{\rm{N}}_r} = \Gamma _{{\rm{N}}_r\uparrow }=\Gamma _{{\rm{N}}_r\downarrow }$, and use the normal lead band half-width *D* as convenient unit for the energies, $D \equiv 1$.

The perspectives of using the Majorana modes for quantum computation rely on their fault-tolerant nature resulting from the topological protection. To guarantee such protection, the nanowire must be safely longer than the superconducting coherence length, such that the overlap between the Majorana edge modes ($\epsilon _M$) is negligible. This constraint, however, is hardly satisfied in short nanowires. Its influence on hybrid structures with a single quantum dot has been recently considered in Refs.^20,29,32–35^. Here, we extend these considerations to the setup with the second quantum dot defined at the other end of the wire, to allow for nonlocal measurements.

## Results

In experimental realizations of our hybrid structure (Fig. 1), the quantum dots and topological nanowire are usually deposited directly on (or covered by) superconducting substrate, while the metallic STM tip is placed in some vicinity of the quantum dots. It is hence reasonable to expect that the coupling of ${\rm{QD}}_1$ to the superconductor is considerably stronger than the hybridization of individual dots with the external metallic electrodes. Therefore, for specific computations we assume the following couplings $\Gamma _S=0.1$ and $\Gamma _{N_1}=\Gamma _{N_2}=0.01$. To account for a short topological nanowire, we also impose a finite overlap between the Majorana modes, $\varepsilon _M=0.05$.

In what follows, we present the (normalized) spectral functions of both quantum dots

7 $$\begin{aligned} A_{r\sigma }(\omega )= -\Gamma _{N_r} \text{ Im } \langle \!\langle d_{r\sigma };d_{r\sigma }^{\dag }\rangle \!\rangle _{\omega +i0^{+}}, \end{aligned}$$

where $\langle \!\langle d_{r\sigma };d_{r\sigma }^{\dag }\rangle \!\rangle _{\omega +i0^{+}}$ is the single particle Green’s function obtained exactly from the equation of motion technique (see the Methods section for details). Focusing on the nonlocal effects, we neglect the Coulomb repulsion, however, qualitative effects of such interactions are briefly discussed in Discussion section. In the following subsections we consider two qualitatively-different situations:

(i) The *local setup*, where the topological segment is long enough to prohibit a direct tunneling of electrons from the given quantum dot to the opposite-side Majorana mode. In other words, in this scenario each quantum dot is assumed to be coupled only to its neighbouring edge state, i.e. $t'_{1}=t'_{2}=0$ and thus $\lambda _{1\sigma }^R = \lambda _{2\sigma }^L = 0$.
(ii) The *nonlocal setup*, in which the electrons can be exchanged between the quantum dots and both boundary Majorana modes, but their couplings are pretty asymmetric. It is reasonable to assume that even for a very short wire, the tunnel coupling between each quantum dot and its neighbouring edge state is much stronger than the coupling to the edge state on the opposite side of the chain. We thus assume $t_{1}=t_{2}=0.05$, unless stated otherwise, and study how the system’s characteristics depend on the coupling of each quantum dot to the corresponding distant Majorana mode, $t'_{1}=t'_{2}\ne 0$.

### The case of local correlations

#### Quasiparticle states and spectral behavior

**Figure 2:**
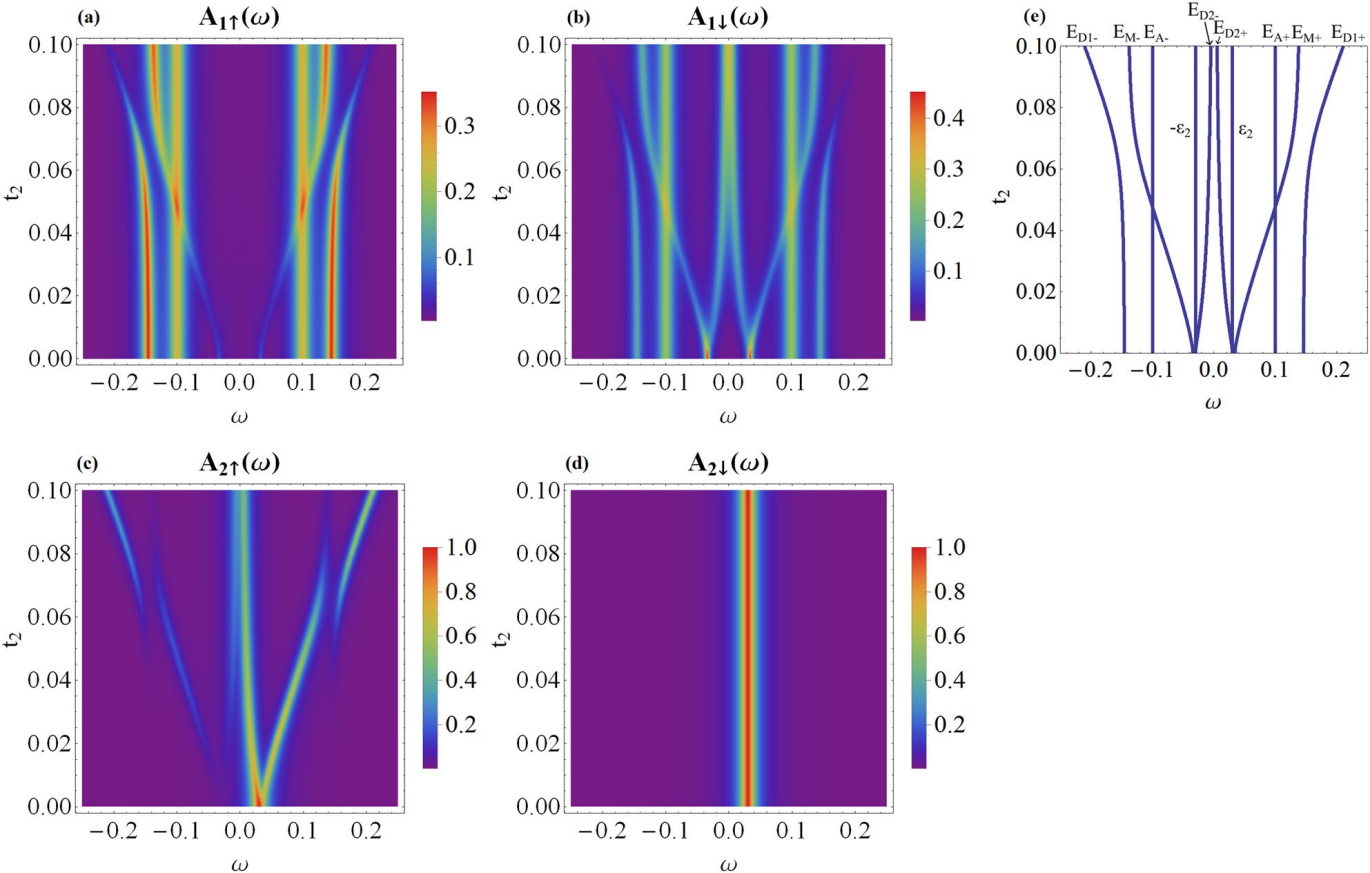
The energy dependence of the spin-resolved spectral function of QD_1_ (**a** and **b** panels) and QD_2_ (**c** and **d** panels) versus the coupling $t_{2}$. Results are obtained for the following model parameters: $\epsilon _1=0$, $\epsilon _2=0.03$, $\varepsilon _M=0.05$, $t_{1}=0.05$ and $\theta =\pi$. Panel (**e**) displays the quasiparticle energies $E_{M\pm }$, $E_{D1\pm }$ and $E_{D2\pm }$ obtained for $\Gamma _{N1}=0=\Gamma _{N2}$.

To begin with, let us analyze the local case, i.e. when quantum dots are directly coupled only to the neighboring Majorana modes. It is instructive to investigate the specific situation when only QD_1_ is coupled to MW ($t_{1}\ne 0$), ignoring any coupling of QD_2_ to the topological superconductor ($t_{2} = 0$). Under such conditions, an interplay of the on-dot pairing and the leakage of the Majorana mode gives rise to emergence of 3 pairs of quasiparticles. Two of them occur at $E_{A\pm }=\pm E_{A}$, where $E_A=\sqrt{\epsilon _1^2+\Gamma _S^2}$, and represent the conventional Andreev bound states^27,36,37^. The additional quasiparticles at energies

8 $$\begin{aligned} E^0_{M1\pm }= & {} \pm \sqrt{A-sgn(E_A-\epsilon _M)\sqrt{A^2-\epsilon _M^2 E_A^2}}, \end{aligned}$$

9 $$\begin{aligned} E^0_{D1\pm }= & {} \pm \sqrt{A+sgn(E_A-\epsilon _M)\sqrt{A^2-\epsilon _M^2 E_A^2}}, \end{aligned}$$

where $A=2t_{1}^2+\frac{\epsilon _M^2+E_A^2}{2}$, originate from the Majorana modes and depend on the overlap $\epsilon _M$ and *sgn*(*x*) is signum function. For a long topological chain ($\epsilon _M \rightarrow 0$), two of these quasiparticle states (8) merge into a zero-energy level, $E_{M1 \pm }=0$. Then, the spectrum of QD_1_ consists of: 2 usual Andreev states, 2 Andreev-Majorana hybrid quasiparticles and the (doubly degenerate) zero-energy mode. Due to the superconducting proximity effect, all these quasiparticle states contribute to the spectral density of QD_1_, though with different spectral weights depending on spin, cf. Fig. 2a,b for $t_2=0$. Some more details about this Majorana leakage in the limit of $\epsilon _M\rightarrow 0$ have been discussed in Ref.^37^.

In the opposite case, when only QD_2_ is coupled to the topological nanowire ($t_{2} \ne 0$) and QD_1_ is absent ($t_{1} = 0$), one obtains 5 quasiparticle states. Their character, however, is very different from the former situation, due to the absence of the Andreev bound states. The ground state of QD_2_ occurs near $\epsilon _2$, which is neither affected by $t_{2}$ nor by $\epsilon _M$. Its hole-counterpart at $-\epsilon _2$ has no spectral weight in the absence of conventional superconducting lead. The remaining Majorana-like $E^0_{M2\pm }$ and dot-like $E^0_{D2\pm }$ quasiparticle levels emerge at

10 $$\begin{aligned} E^0_{M2\pm }= & {} \pm \sqrt{B-sgn(\epsilon _2-\epsilon _M)\sqrt{B^2-\epsilon _M^2 \epsilon _2^2}}, \end{aligned}$$

11 $$\begin{aligned} E^0_{D2\pm }= & {} \pm \sqrt{B+sgn(\epsilon _2-\epsilon _M)\sqrt{B^2-\epsilon _M^2 \epsilon _2^2}}, \end{aligned}$$

where $B=2t_{2}^2+\frac{\epsilon _M^2+\epsilon _2^2}{2}$, in analogy to Eqs. (8) and (9).

For $t_{1} \ne 0$ and $t_{2} \ne 0$, the canting intertwines the spectra of QD_1_ and QD_2_, even when the Majorana mode couples only to the spin-$\uparrow$ channel ($\theta =\pi$), as presented in Fig. 2 for $t_2 > 0$. The quasiparticles at QD_1_ include two Andreev-type bound states $E_{A\pm }$ independently of the coupling of QD_2_ to MW. Similarly, for QD_2_, we observe the state at $\epsilon _{2}$, no matter what the coupling of QD_1_ to topological superconductor is. The remaining quasiparticles arise from the hybridization of QD_1_ and QD_2_ states with the Majorana mode ($E_{M\pm }$, $E_{D1\pm }$ and $E_{D2\pm }$).

**Figure 3:**
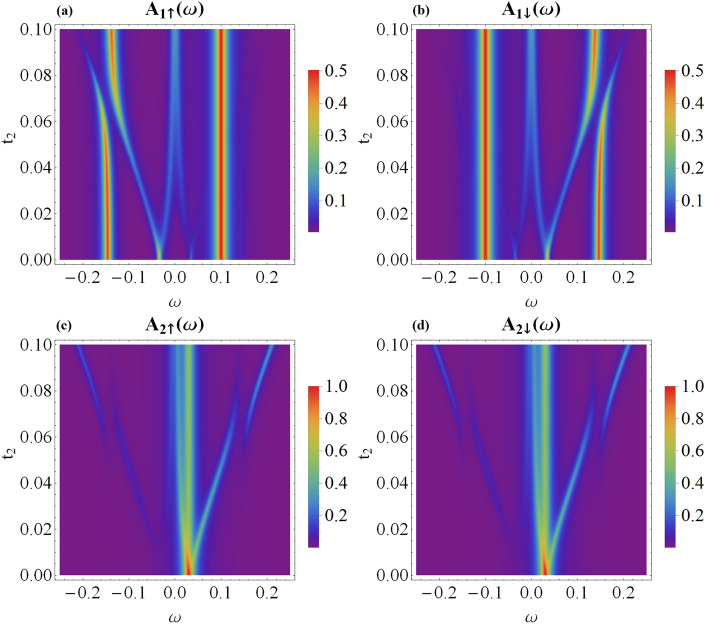
Variation of the spin-dependent spectral functions of QD_1_ (**a** and **b** panels) and QD_2_ (**c** and **d** panels) with respect to the coupling $t_{2}$ calculated for the same model parameters as in Fig. 2 assuming the spin canting angle $\theta =\frac{\pi }{2}$. The quasiparticle energies $E_{M\pm }$, $E_{D1\pm }$ and $E_{D2\pm }$ coincide with those presented in Fig. 2 (**e**).

Figure 2, by presenting the spin-resolved spectral functions of individual quantum dots, $A_{r\sigma }(\omega )$, reveals the influence of the coupling $t_{2}$ between QD_2_ and the Majorana mode on the energy spectrum of QD_1_ and QD_2_, respectively. In $A_{1 \uparrow }(\omega )$ its effect relies mainly on shifting the energy levels of the hybrid quasiparticles. More interesting results can be seen due to the relationship between the energy spectrum of spin-$\downarrow$ electrons of QD_1_ and spin-$\uparrow$ electrons of QD_2_. If $t_2$ is non-vanishing, one of the hybrid states in $A_{1 \downarrow }(\omega )$ splits to form the high and low energy branches. In total, we obtain 8 quasiparticle states associated with: 2 ABSs and 6 hybrid states. The molecular states appearing in $A_{2 \uparrow }(\omega )$ coincide with the energies of molecular states in $A_{1 \downarrow }(\omega )$. Such result indicates a mutual origin of the opposite spin electrons at the distant quantum dots transmitted through the Majorana modes. Moreover, Fig. 2b clearly shows that $A_{1\downarrow }(\omega )$ is indirectly affected by the topological superconductor even when only the spin-$\uparrow$ electrons are directly coupled to the wire. This is caused by the on-dot pairing induced by the superconducting lead^27^. On the contrary, the spectral function $A_{2\downarrow }(\omega )$ is then unaffected by the presence of the Majorana modes, cf. Fig. 2d.

We now consider the case when the canting leads to noncolinearity by setting $\theta =\frac{\pi }{2}$, cf. Eqs. (5) and (6). The corresponding spin-dependent spectral functions for this situation are presented in Fig. 3. First of all, one can clearly notice that the spectrum of QD_1_ is characterized by $A_{1\uparrow }(\omega ) = A_{1\downarrow }(-\omega )$. Furthermore, $A_{1\sigma }(\omega )$ reveals only a single Andreev peak, at $\omega = E_{A+}$ for spin-$\uparrow$ and at $\omega =E_{A-}$ for spin-$\downarrow$ sectors, respectively. Additionally, in both spectral functions $A_{1\sigma }(\omega )$ we recognize the quasiparticle peaks at $E_{M\pm }$ originating from QD_2_.

The symmetry of the spin-resolved spectral functions $A_{1\sigma }(\omega )$ displayed in Fig. 3 originates partly from the canting angle $\theta =\pi /2$ and partly from the superconducting proximity effect, which mixes the particle with hole degrees of freedom. Physically it means that the spin-$\uparrow$ electrons ($\omega <0$) are mixed with spin-$\downarrow$ holes ($\omega >0$). Similar effect occurs between $\omega <0$ spin-$\downarrow$ and $\omega >0$ spin-$\uparrow$ quasiparticle states of QD_1_. Concerning the spectral behavior of QD_2_, we observe that $A_{2\uparrow }(\omega )=A_{2\downarrow }(\omega )$, and in both spin sectors there are present the quasiparticles at $\omega =E_{D1\pm }$, transmitted from QD_1_ via the topological superconducting nanowire.

#### Andreev transmission

Junctions consisting of metallic and superconducting leads allow for the anomalous electron-to-hole (Andreev) scattering mechanism, active particularly in the subgap regime. In the N_1_-QD_1_-SC circuit we can observe the direct Andreev reflection (DAR) processes, when the incoming electron from the metallic lead is scattered as a hole back to the same electrode. In addition, the side-attached topological superconductor allows for the non-local crossed Andreev reflection (CAR), in which a hole is nonlocally transmitted to the electrode on the right hand side of the wire^38–41^. The transmission coefficients for the direct and crossed Andreev refection processes can be expressed by the off-diagonal terms of the matrix Green’s functions

12 $$\begin{aligned} T_r^{DAR}(\omega )=\sum _{\sigma }\Gamma _{N_r\sigma }\Gamma _{N_r\bar{\sigma }}\left| \langle \!\langle d_{r\sigma };d_{r\bar{\sigma }}\rangle \!\rangle _\omega \right| ^{2}, \end{aligned}$$

and

13 $$\begin{aligned} T^{CAR}(\omega )=\sum _{\sigma }\big (\Gamma _{N_1\sigma }\Gamma _{N_2\bar{\sigma }}\left| \langle \!\langle d_{1\sigma };d_{2\bar{\sigma }}\rangle \!\rangle _\omega \right| ^{2}+\Gamma _{N_2\sigma }\Gamma _{N_1\bar{\sigma }}\left| \langle \!\langle d_{2\sigma };d_{1\bar{\sigma }}\rangle \!\rangle _\omega \right| ^{2}\big ). \end{aligned}$$

**Figure 4:**
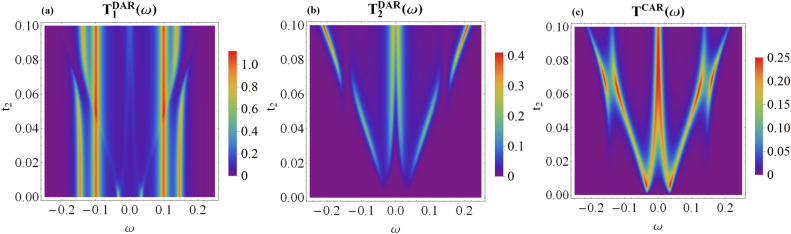
The transmittance of the DAR and CAR tunneling channels plotted versus energy $\omega$ and the coupling $t_{2}$ assuming the spin canting angle $\theta =\frac{\pi }{2}$ and the other parameters the same as in Fig. 2.

Figure 4 displays the transmittance obtained for the direct (local) and crossed (nonlocal) Andreev tunneling. In the low-temperature limit, the differential tunnelling conductance would be a product of the transmittance and conductance quantum $4e^{2}/h$. By inspecting the DAR channel, we find signatures of all quasiparticle states induced in our hybrid structure. For $\theta =\pi$, the second quantum dot neither reveals the direct nor crossed particle-to-hole reflections because superconducting proximity effect is absent. For $\theta =\pi /2$ (as well as for other angles different from multiples of $\pi$) we clearly see the nonlocal (CAR) reflections and even the direct Andreev scattering in the N_2_-QD_2_-MW setup.

### Nonlocal correlations

#### Spectral behavior and Andreev transmittance

**Figure 5:**
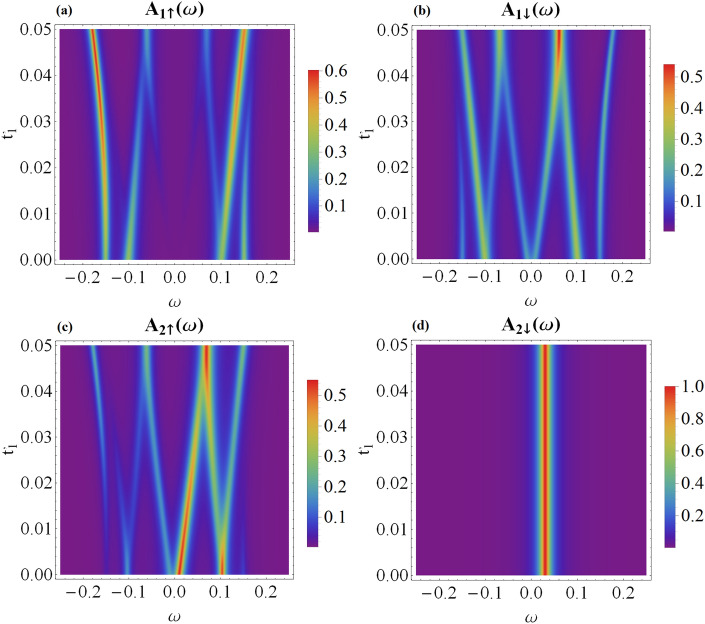
Evolution of the spin-resolved spectra of QD_1_ (panels a, b) and QD_2_ (panels c, d) obtained for finite nonlocal couplings $t'_{1}=t'_{2}$. The parameters are the same as in Fig. 2 with $t_{1}=t_{2}=0.05$.

Let us now focus on the case when the quantum dots side-attached to the short topological superconductor hybridize with both boundary Majorana modes, $t'_{r}\ne 0$, and the ratio $\eta ^2=\left| t'_{r}/t_{r}\right|$ can be regarded as a qualitative measure of nonlocality in this configuration^20,29,33,42^. Figure 5a,b show the electronic spectrum of QD_1_, while varying with respect to the nonlocal coupling $t'_{1}=t'_{2}$. We notice substantial influence on the Andreev states $E_A$ accompanied by redistribution of their spectral weights. In particular, the quasiparticle state at $E_{A-}$ of the spin-$\uparrow$ sector transmits its spectral weight to the state at $E_{D1-}$. Similarly, in the spin-$\downarrow$ sector the spectral weight of $E_{A+}$ is transferred to $E_{D1+}$. Furthermore, we observe additional changes in the spectrum of QD_1_ appearing at energies $E_{D2\pm }$, originating from QD_2_ transmitted via the short topological nanowire.

**Figure 6:**
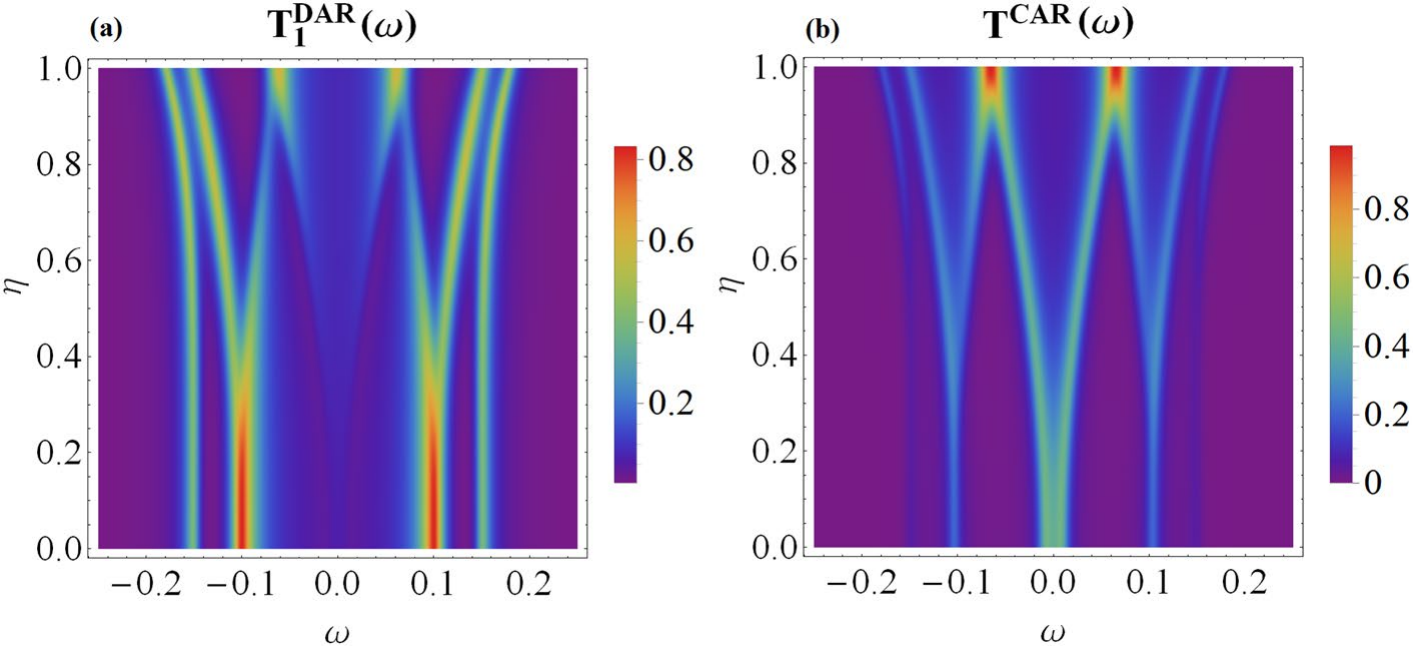
Transmittance of the DAR and CAR transport channels versus energy $\omega$ and the MBS nonlocality parameter $\eta =\sqrt{\left| t'_{r}/t_{r}\right| }$. Results are obtained for the same parameters as in Fig. 5.

The spin-resolved spectral functions of QD_2_, revealing the corresponding quasiparticle states, are shown in Fig. 5c,d. For the colinear case, $\theta =\pi$, we clearly see that only spin-$\uparrow$ sector of QD_2_ exhibits the same quasiparticle states as QD_1_. The opposite spin is completely unaffected, as manifested by a single peak at $\varepsilon _{2}$. Such a behavior indicates absence of any on-dot pairing at QD_2_. For other values of $\theta$, the spin-resolved spectral function of QD_2_ shows signatures of the conventional quasiparticle states ($E_{D1\pm }$) and Majorana-type features ($E_{M\pm }$) acquired from QD_1_ via the short topological superconductor. For $t'_{1} \ne 0$, the Andreev bound states become mixed with the quasiparticle states $E_{D1\pm }$, therefore weak superconducting correlations are indirectly induced at QD_2_.

Figure 6 presents the signatures of the quasiparticle states possible to be probed by the Andreev spectroscopy. Specifically, we show variation of the direct and crossed Andreev transmittance with respect to $\eta =\sqrt{\left| t'_{r}/t_{r}\right| }$ obtained for the symmetric couplings $t_1=t_2$ and $t'_{1}=t'_{2}$. We notice that the local Andreev scattering (DAR) is a particle-hole symmetrized version of the spin-resolved spectral functions of QD_1_ [see panel a and b in Fig. 5]. The nonlocal (CAR) transmittance, on the other hand, mixes the particle with hole degrees of freedom between the distant quantum dots. Such effect is partly caused by the overlap of Majorana modes, $\epsilon _{M}\ne 0$, and additionally comes from the nonlocal hopping, $t'_{r}\ne 0$.

#### Singlet and triplet electron pairing

**Figure 7:**
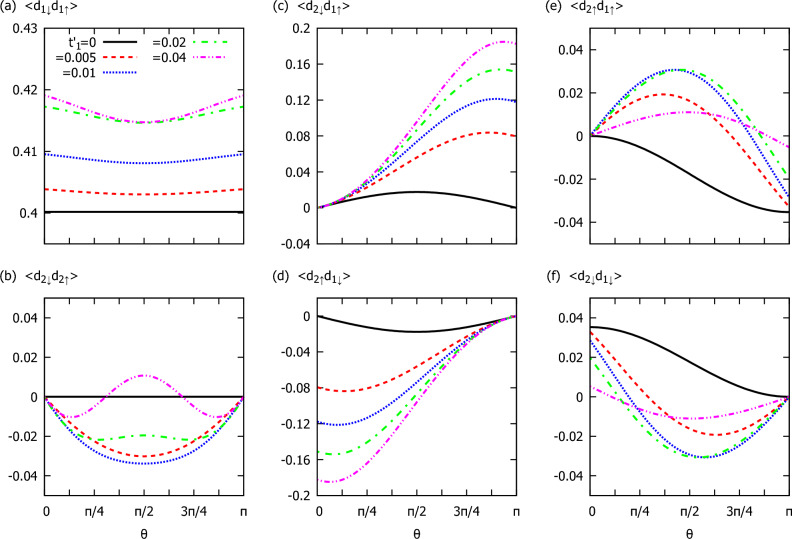
Various channels of the electron pairing induced at individual quantum dots and nonlocally between the dots versus the spin canting angle, $\theta$. Results are obtained at zero temperature for several values of the coupling $t'_{1}=t'_{2}$ (as indicated), assuming the following model parameters: $\epsilon _1=\epsilon _2=0$, $\varepsilon _M=0.05$ and $t_{1}=t_{2}=0.05$.

To characterize the efficiency of the superconducting proximity effect we have calculated the expectation values of the on-dot electron pairings, which (at zero temperature) are defined by

14 $$\begin{aligned} \langle d_{r\downarrow }d_{r\uparrow }\rangle =-\frac{1}{\pi }\int \limits _{-\infty }^0{\text{ Im } \langle \!\langle d_{r\uparrow };d_{r\downarrow }\rangle \!\rangle _\omega \; d\omega }. \end{aligned}$$

In addition, we have also studied the nonlocal electron pairs induced between the quantum dots, considering the singlet and triplet channels^43^. For the local situation, $t_{i}'=0$, the local pairing induced at QD_1_ is insensitive to the spin canting angle, whereas the on-dot pairing of QD_2_ is absent for all values of $\theta$. In contrast to this, for $t'_{1}\ne 0$, the Andreev bound states $E_{D1\pm }$ appear simultaneously in both quantum dots, though with different amplitudes. The local pairings depend then on both $t'_{1}$ and the spin canting angle $\theta$. In Fig. 7a we present the dependence of the local pairing $\langle d_{1\downarrow }d_{1\uparrow }\rangle$ on $\theta$ for several values of the hopping $t'_{1}=t'_{2}$, as indicated. Optimal conditions for this pairing coincide with the colinear cases ($\theta =0$ or $\theta =\pi$) and the minimal local pairing occurs at perpendicular canting, $\theta =\pi /2$. Figure 7b illustrates the variation of the on-dot pairing of QD_2_ with respect to $\theta$. In the weak coupling limit (small $t'_{1}$), the local electron pairing induced in QD_2_ is negative and its minimum occurs at $\theta =\pi /2$. On the other hand, in the strong coupling limit (for large $t'_{1}$), such indirectly induced electron pairing changes sign and its maximum occurs again at $\theta =\pi /2$. For the colinear cases ($\theta =0$ or $\theta =\pi$), the local pairing of QD_2_ vanishes, $\langle d_{2\downarrow }d_{2\uparrow }\rangle =0$, no matter what the value of $t'_{1}$ is.

To summarize this section, we emphasize that the short topological superconductor with a finite overlap between the Majorana modes ($\epsilon _M \ne 0$) enables crossed Andreev reflections, originating from the nonlocal electron pairing $\langle d_{2\sigma }d_{1\sigma '}\rangle$. For the colinear cases, we observe vanishing of the nonlocal correlations $\langle d_{2 \uparrow } d_{1 \uparrow } \rangle$ and $\langle d_{2 \downarrow } d_{1 \uparrow } \rangle$ (for $\theta =0$, i.e. no spin canting) or pairings $\langle d_{2 \uparrow } d_{1 \downarrow } \rangle$ and $\langle d_{2 \downarrow } d_{1 \downarrow } \rangle$ (for $\theta =\pi$, i.e. spin quantization axis reversal between the wire ends). Otherwise, all the inter-dot electron pairs survive, allowing for the nonlocal Andreev scattering. Detailed dependence of the inter-dot (singlet and triplet) pairing functions against the spin canting angle ($\theta$) is displayed in Fig. 7c–f.

## Discussion

We have investigated the quasiparticle spectrum of the hybrid structure formed of two quantum dots interconnected through a short topological superconducting nanowire, hosting the overlaping Majorana boundary modes. We have found clear signatures of nonlocal (inter-dot) correlations in spectral functions of the system, which would be feasible for detection with STM techniques. Their precise form depends on the magnitude of the dots coupling to the boundary modes of the topological wire. For the case when quantum dots are coupled only to the adjacent edges of the wire, we predict the following manifestations of nonlocality.

(i) In maximally spin-canted case ($\theta =\pi$), the spin-$\downarrow$ QD_1_ spectrum gains contributions from all the quasiparticles visible in the spin-$\uparrow$ QD_2_ spectrum, cf. Fig. 2. This can be seen as transmission of the quasiparticle weight through the Majorana modes. At the same time, only some of the QD_1_ quasiparticles are admitted to QD_2_, which is best illustrated by the spin-$\downarrow$ QD_2_ spectrum, unaffected by coupling to the wire.
(ii) For generic canting, $\theta \ne 0$ and $\theta \ne \pi$, the complete set of the molecular quasiparticles are apparent in the spin-resolved speactral functions of both quantum dots.

In more general case, when the dots are coupled to both Majorana modes ($t'_1=t'_2\ne 0$), the inter-dot electron pairs are formed, both in the singlet and triplet channels. Such nonlocal pairs can be experimentally detected by the crossed Andreev reflection spectroscopy, using either the unpolarized or spin-polarized external electrodes.

In the present work we consider the case of quantum dots energy levels close to the Fermi energy. For further in-depth study it would be useful to take into account the on-site Coulomb repulsion between the opposite-spin electrons. This would open the way to address also Coulomb blockade and Kondo regimes. Previous studies of the superconducting nanohybrid structures indicated that the strong Coulomb repulsion could also suppress the local pairing potential. For this reason one might expect the correlations to predominantly affect the quaspiarticle energies of the bound states at QD_1_. As regards the second quantum dot, the correlations would give rise to the Zeeman term^44,45^. Additionally, in both quantum dots renormalization of the effective spin exchange interaction would occur, playing essential role in the low-temperature Kondo regime, when the quantum dots are approximately half-filled. These issues, however, are beyond the scope of the present study, which is focused on the nonlocal pairing correlations relevant for the crossed Andreev spectroscopy.

Let us finally comment on possible experimental means of verifying our predictions. Dvir et al.^21^ have provided experimental evidence for the realization of the Kitaev chain composed of just two sites. In practice, these sites represented pieces of the semiconducting nanowire with discrete energy levels brought in contact via the conventional superconductor. Depending on the energy levels (tunable by gate potentials) and affected by the spin-orbit and Zeeman effect these sites were able to approach sweet points, inducing the Majorana modes. Since they were spatially close to one another, their wave functions might have been hybridized ($\epsilon _{M}\ne 0$). Our present study could be relevant to this situation with additional quantum dots attached on opposite sides. Upon forming the circuits with external electrodes, the quasiparticle states of these attached quantum dots can be probed experimentally. For low bias voltages (smaller than the energy gap of superconductor), the only transport channel would by contributed by the particle-to-hole (Andreev) scattering. Our estimations of the Andreev transmittance (corresponding roughly to the differential Andreev conductance at low temperatures) would thus enable exploring the direct and crossed Andreev conductance, revealing the local and nonlocal cross-correlations transmitted between these dots via the overlapping Majorana modes.

## Methods

To study the hybrid structure shown in Fig. 1, we have determined the quasiparticle states. We have taken into account the leakage of Majorana modes into both quantum dots and the superconducting proximity effect, the latter inducing conventional Andreev bound states at QD_1_. Focusing on the subgap energy region, $|\omega | \ll \Delta _{S}$, we simplified the considerations by treating $\Delta _{S}$ as the largest energy scale. In the limit of $\Delta _S \rightarrow \infty$, the fermionic degrees of freedom of the superconducting lead can be integrated out, giving rise to the on-dot electron pairing $- \Gamma _{S} (d_{1 \uparrow }^\dag d_{1 \downarrow }^\dag + d_{1 \downarrow } d_{1 \uparrow })$. Under such circumstances the “proximitized” QD_1_ can be modeled by^24–27^

15 $$\begin{aligned} H_{\rm{L}} = \sum _{\sigma } \varepsilon _{1\sigma } d_{1\sigma }^\dag d_{1\sigma } - \Gamma _{S} (d_{1 \uparrow }^\dag d_{1 \downarrow }^\dag + d_{1 \downarrow } d_{1 \uparrow })+\sum _{{\textbf{k}}\sigma }\varepsilon _{{N_1}{\textbf{k}}} c^\dag _{{N_1}{\textbf{k}}\sigma } c_{{N_1}{\textbf{k}}\sigma } +\sum _{{\textbf{k}}\sigma } V_{{N_1}\sigma } \left( d^\dag _{1 \sigma } c_{{N_1}{\textbf{k}}\sigma } + c^\dag _{{N_1}{\textbf{k}}\sigma } d_{1 \sigma } \right) . \end{aligned}$$

It is also convenient to recast the Majorana operators by the usual fermion operators, $\eta _L = (f^\dag +f)/\sqrt{2}$ and $\eta _R = i(f^\dag -f)/\sqrt{2}$. In this representation, the term (4) of the model Hamiltonian can be expressed as

16 $$\begin{aligned} H_{\rm{MW}} = \sum _{r=1,2}\sum _\sigma \left[ t_{r\sigma }^+\left( d_{r\sigma }^\dag f^\dag +f d_{r\sigma }\right) +t_{r\sigma }^-\left( d_{r\sigma }^\dag f+f^\dag d_{r\sigma }\right) \right] +\varepsilon _M\left( f^\dag f-\frac{1}{2}\right) , \end{aligned}$$

with the spin-dependent hopping integrals

17 $$\begin{aligned} \begin{array}{cc} t_{1\uparrow }^+=t_{1}\sin \frac{\theta _L}{2}+t'_{1}\sin \frac{\theta _R}{2}, &{}t_{1\uparrow }^-=t_{1}\sin \frac{\theta _L}{2}-t'_{1}\sin \frac{\theta _R}{2}, \\ t_{1\downarrow }^+=-t_{1}\cos \frac{\theta _L}{2}+t'_{1}\cos \frac{\theta _R}{2}, &{}t_{1\downarrow }^-=-t_{1}\cos \frac{\theta _L}{2}-t'_{1}\cos \frac{\theta _R}{2}, \end{array} \end{aligned}$$

and

18 $$\begin{aligned} \begin{array}{cc} t_{2\uparrow }^+=t'_{2}\sin \frac{\theta _L}{2}+t_{2}\sin \frac{\theta _R}{2}, &{}t_{2\uparrow }^-=t'_{2}\sin \frac{\theta _L}{2}-t_{2}\sin \frac{\theta _R}{2}, \\ t_{2\downarrow }^+=-t'_{2}\cos \frac{\theta _L}{2}+t_{2}\cos \frac{\theta _R}{2}, &{} t_{2\downarrow }^-=-t'_{2}\cos \frac{\theta _L}{2}-t_{2}\cos \frac{\theta _R}{2}. \end{array} \end{aligned}$$

The influence of the topological nanowire on the side attached quantum dots can be analyzed within the Green’s function approach represented in the particle-hole matrix notation, $\hat{\mathscr{G}}(\omega )= \langle \!\langle \Psi ;\Psi ^\dag \rangle \!\rangle _{\omega }$, with the Nambu spinor defined as $\Psi = (d_{1\uparrow },d_{1\uparrow }^\dag ,d_{1\downarrow },d_{1\downarrow }^\dag ,d_{2\uparrow },d_{2\uparrow }^\dag ,d_{2\downarrow },d_{2\downarrow }^\dag ,f,f^\dag )$. The equation of motion

19 $$\begin{aligned} (\omega +i 0^{+}) \langle \!\langle \Psi _i;\Psi _j\rangle \!\rangle _\omega = \left\langle \left[ \Psi _i,\Psi _j\right] _+ \right\rangle + \langle \!\langle \left[ \Psi _i,H\right] _-;\Psi _j\rangle \!\rangle _\omega \end{aligned}$$

yields the following retarded Green’s function

20 $$\begin{aligned} {\mathscr {G}}^{-1}(\omega ) =\omega \hat{I}+ \left( \begin{array}{cccccccccc} -\tilde{\epsilon }_{1\uparrow } &{}0&{}0&{} \Gamma _{S}&{} 0 &{} 0&{} 0 &{} 0 &{} -t^{-}_{1\uparrow } &{} -t^{+}_{1\uparrow }\\ 0&{}(\tilde{\epsilon }_{1\uparrow })^{*}&{}-\Gamma _{S}&{} 0 &{} 0 &{} 0&{} 0 &{} 0&{} t^{+}_{1\uparrow }&{} t^{-}_{1\uparrow }\\ 0&{}-\Gamma _{S}&{}-\tilde{\epsilon }_{1\downarrow }&{} 0 &{} 0 &{} 0&{} 0 &{} 0&{}-t^{-}_{1\downarrow } &{} -t^{+}_{1\downarrow }\\ \Gamma _{S}&{}0&{}0&{}(\tilde{\epsilon }_{1\downarrow })^* &{} 0 &{} 0&{} 0 &{} 0&{} t^{+}_{1\downarrow } &{} t^{-}_{1\downarrow }\\ 0 &{} 0&{} 0 &{} &{}-\tilde{\epsilon }_{2\uparrow }&{}0&{}0&{} 0&{}-t^{-}_{2\uparrow } &{} -t^{+}_{2\uparrow } \\ 0 &{} 0&{} 0 &{} 0&{}0&{}(\tilde{\epsilon }_{2\uparrow })^*&{}0&{} 0&{} t^{+}_{2\uparrow } &{} t^{-}_{2\uparrow }\\ 0 &{} 0&{} 0 &{} 0&{}0&{}0&{}-\tilde{\epsilon }_{2\downarrow }&{} 0&{} -t^{-}_{2\downarrow } &{} -t^{+}_{2\downarrow }\\ 0&{} 0&{} 0 &{} 0&{}0&{}0&{}0&{}(\tilde{\epsilon }_{2\downarrow })^*&{}t^{+}_{2\downarrow } &{} t^{-}_{2\downarrow } \\ -t^{-}_{1\uparrow } &{} t^{+}_{1\uparrow } &{}-t^{-}_{1\downarrow } &{} t^{+}_{1\downarrow }&{} -t^{-}_{2\uparrow } &{} t^{+}_{2\uparrow } &{}-t^{-}_{2\downarrow } &{} t^{+}_{2\downarrow }&{} -\epsilon _{M}&{} 0\\ -t^{+}_{1\uparrow }&{} t^{-}_{1\uparrow } &{}-t^{+}_{1\downarrow } &{} t^{-}_{1\downarrow }&{} -t^{+}_{2\uparrow } &{} t^{-}_{2\uparrow } &{}-t^{+}_{2\downarrow } &{} t^{-}_{2\downarrow }&{} 0 &{} \epsilon _{M} \end{array}\right) , \end{aligned}$$

where $\hat{I}$ stands for the identity matrix and $\tilde{\epsilon }_{i\sigma }=\epsilon _{i\sigma }-i\Gamma _{N_i}$. We have used various parts of this matrix Green’s function for computing the spin-resolved spectral functions of both quantum dots, the direct and crossed Andreev transmittances as well as for evaluation of the local and nonlocal pairing functions.

## Influence of Zeeman field

Topologically nontrivial superconductivity of semiconducting nanowires arises upon combining the Rashba interaction with the superconducting proximity effect in the presence of sufficiently strong external magnetic field (usually on the order of 1 T). The same criterion is necessary for the minimally short-length topological nanowires^21,22^, where the Majorana modes might overlap between themselves. For this reason, it is important to check the influence of magnetic field on the quasiparticle spectra of our hybrid structure (Fig. 1), taking into account the Zeeman splitting of the quantum dot levels, $B=\varepsilon _{i\downarrow }-\varepsilon _{i\uparrow }$.

**Figure 8:**
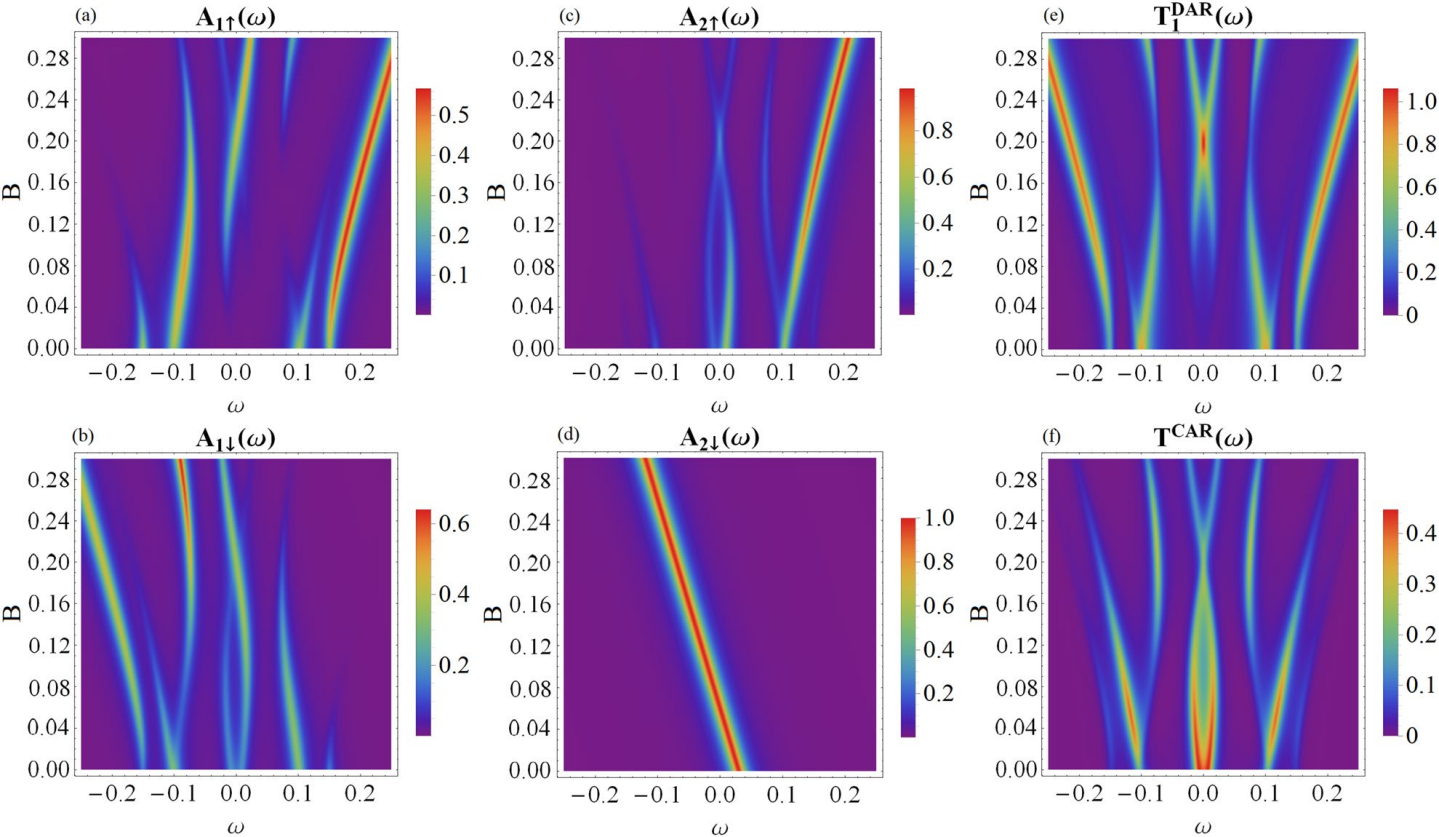
The spin-dependent spectral functions of QD_1_ (**a** and **b** panels) and QD_2_ (**c** and **d** panels) and the transmittance of the DAR (**e**) and CAR (**f**) tunneling channels with respect to the Zeeman field *B*. The results are obtained for the model parameters: $\epsilon _1=0$, $\epsilon _2=0.03$, $\varepsilon _M=0.05$, $t_{1}=t_{2}=0.05$, $t'_{1}=t'_{2}=0$, and $\theta =\pi$.

Similar issue has been previously addressed by Prada et al.^29^ for a single quantum dot attached to the short topological wire, predicting either bowtie-like (for $t_{1}' \ll \epsilon _{M}, t_{1}$) or diamond-like (for $\epsilon _{M} \ll t_{1}, t_{1}'$) superstructure of the quasi-Majorana modes, respectively. For brevity, we focus here on the linear configuration ($t_{1}'=0=t_{2}'$) and analyze evolution of the spin-resolved quasiparticles of the quantum dots with respect to the Zeeman field *B* (see Fig. 8). In close analogy to the results reported in Refs^29,35^ we obtain the bowtie feature in the spin-$\uparrow$ spectral functions of both quantum dots. Furthermore, we notice this bowtie-like shape appearing in the spin-$\downarrow$ spectral function of QD_1_, which is indirectly driven by the on-dot pairing between the opposite spins. Such bowtie-structure is in turn evident, both in the direct and crossed Andreev transmittance (see right h.s. panels in Fig. 8), thus it would be detectable experimentally. As far as the higher-energy (trivial) quasiparticles are concerned, they reveal rather complex variation against the Zeeman field, depending on various spin arrangements of the quantum dots and additionally being affected by the on-dot pairing at QD_1_ (discussed at length in the main part of our manuscript).

## Data Availability

The datasets generated and analyzed during the current study are available from the repository^46^ and/or upon request from the corresponding author.
